# Supplementary material for: Polyketide synthase gene clusters in the ericoid mycorrhizal fungus Oidiodendron maius: genome-wide prediction, expression profiling, and functional characterization of knockout mutants
Source: Front Plant Sci. 2026 Sep 16;17:1924430. doi: 10.3389/fpls.2026.1924430 (PMC13623903; doi:10.3389/fpls.2026.1924430)
Supplement: Supplementary file 1 [file SupplementaryFile1.zip › Supplementary Materials/Supplementary Folder 1.zip!/Oidma1_AssemblyScaffolds_Repeatmasked/knownclusterblast/cluster2/jgi.p_Oidma1_46813_mibig_hits.html]

| MIBiG Protein | Description | MIBiG Cluster | MiBiG Product | % ID | % Coverage | BLAST Score | E-value |
| --- | --- | --- | --- | --- | --- | --- | --- |
| AAX98209.1 | 5-aminolevulinate\_synthase | BGC0000052 | ECO-02301\_biosynthetic\_gene\_cluster | 48 | 82.4701195219 | 368 | 1.3e-101 |
| AIL50190.1 | putative\_5-aminolevulinate\_synthase | BGC0000213 | Colabomycin\_biosynthetic\_gene\_cluster | 48 | 82.4701195219 | 365 | 1.1e-100 |
| AGY30678.1 | Ann2 | BGC0001298 | 4-Z-annimycin\_biosynthetic\_gene\_cluster | 49 | 80.6772908367 | 363 | 4.3e-100 |
| ADI58646.1 | AsuD2 | BGC0000187 | Asukamycin\_biosynthetic\_gene\_cluster | 46 | 82.2709163347 | 347 | 3.2e-95 |
| ABJ90148.1 | MoeC4 | BGC0000805 | Moenomycin\_biosynthetic\_gene\_cluster | 47 | 78.8844621514 | 341 | 1.8e-93 |
| ADC79615.1 | BafZ | BGC0000028 | Bafilomycin\_biosynthetic\_gene\_cluster | 47 | 78.2868525896 | 336 | 7.4e-92 |
